# *ARID1A* mutation sensitizes most ovarian clear cell carcinomas to BET inhibitors

**DOI:** 10.1038/s41388-018-0300-6

**Published:** 2018-05-15

**Authors:** Katrien Berns, Joseph J. Caumanns, E. Marielle Hijmans, Annemiek M. C. Gennissen, Tesa M. Severson, Bastiaan Evers, G. Bea A. Wisman, Gert Jan Meersma, Cor Lieftink, Roderick L. Beijersbergen, Hiroaki Itamochi, Ate G. J. van der Zee, Steven de Jong, René Bernards

**Affiliations:** 1 Division of Molecular Carcinogenesis and Oncode Institute, The Netherlands Cancer Institute, Plesmanlaan 121, Amsterdam, 1066 CX The Netherlands; 2 Gynaecologic Oncology, Cancer Research Centre Groningen, University Medical Center Groningen, University of Groningen, Hanzeplein 1, Groningen, 9713 GZ The Netherlands; 3 Department of Obstetrics and Gynaecology, Iwate Medical University School of Medicine, Iwate, Morioka, 020-8505 Japan; 4 Medical Oncology, Cancer Research Centre Groningen, University Medical Center Groningen, University of Groningen, Hanzeplein 1, Groningen, 9713 GZ The Netherlands

## Abstract

Current treatment for advanced stage ovarian clear cell cancer is severely hampered by a lack of effective systemic therapy options, leading to a poor outlook for these patients. Sequencing studies revealed that *ARID1A* is mutated in over 50% of ovarian clear cell carcinomas. To search for a rational approach to target ovarian clear cell cancers with *ARID1A* mutations, we performed kinome-centered lethality screens in a large panel of ovarian clear cell carcinoma cell lines. Using the largest OCCC cell line panel established to date, we show here that BRD2 inhibition is predominantly lethal in *ARID1A* mutated ovarian clear cell cancer cells. Importantly, small molecule inhibitors of the BET (bromodomain and extra terminal domain) family of proteins, to which BRD2 belongs, specifically inhibit proliferation of *ARID1A* mutated cell lines, both in vitro and in ovarian clear cell cancer xenografts and patient-derived xenograft models. BET inhibitors cause a reduction in the expression of multiple SWI/SNF members including *ARID1B*, providing a potential explanation for the observed lethal interaction with *ARID1A* loss. Our data indicate that BET inhibition may represent a novel treatment strategy for a subset of *ARID1A* mutated ovarian clear cell carcinomas.

## Introduction

Epithelial ovarian cancer covers approximately 90% of all ovarian cancers and is the most common cause of mortality in women with gynecologic cancers. Five histological subtypes have been defined for epithelial ovarian cancers: high-grade serous, low-grade serous, clear cell, mucinous, and endometrioid [1, 2]. Although each subtype has unique molecular and clinical features, all epithelial ovarian subtypes are still treated similarly, consisting of de-bulking surgery in combination with platinum-based chemotherapy. Ovarian clear cell carcinoma (OCCC), the second most common subtype, appears to have a worse prognosis than the more common high-grade serous carcinoma, suggesting that current treatments are ineffective for OCCC, especially related to a poor response to platinum-based chemotherapy. Therefore, new treatment strategies for OCCC are urgently needed [3]. Development of OCCC has been linked to endometriosis and is characterized by a high mutation frequency of *ARID1A* (>50%), a subunit of the SWI/SNF chromatin remodeling complex [4, 5]. Given the nature of the mutations it is generally accepted that ARID1A functions as a tumor suppressor gene. The SWI/SNF chromatin-remodeling complex regulates the dynamic repositioning of nucleosomes, therefore the loss of *ARID1A* could globally impact gene expression through deregulated transcription [6]. Since *ARID1A* mutations have been identified in pre-neoplastic lesions it is suspected that ARID1A loss is an early event in the development of OCCC [4]. Possibly, loss of ARID1A activates major signaling pathways that confer an advantage to the tumor cells through enhanced proliferation and/or survival. Given that *ARID1A* is inactivated by mutation in OCCC, we pursued a synthetic lethal screening strategy to identify druggable targets in OCCC. We performed lethal kinome short hairpin (shRNA) screens in the largest panel of OCCC cell lines established to date having different *ARID1A* mutation status. Here, we report the identification of *BRD2*, a member of the BET (bromodomain and extra terminal domain) family, whose knockdown resulted in enhanced toxicity predominantly in *ARID1A* mutant cell lines. Importantly, our data demonstrate enhanced sensitivity of *ARID1A* mutated cells to the BET inhibitors JQ1 and iBET-762. Furthermore, the in vitro drug sensitivity data presented here were validated both in OCCC cell line xenografts and in OCCC patient-derived xenograft (PDX) models. Collectively, our data suggest a new treatment option for *ARID1A* mutant OCCC that warrants clinical exploration.

## Results

### *ARID1A* synthetic lethal screens in OCCC cell line panel

**Fig. 1:**
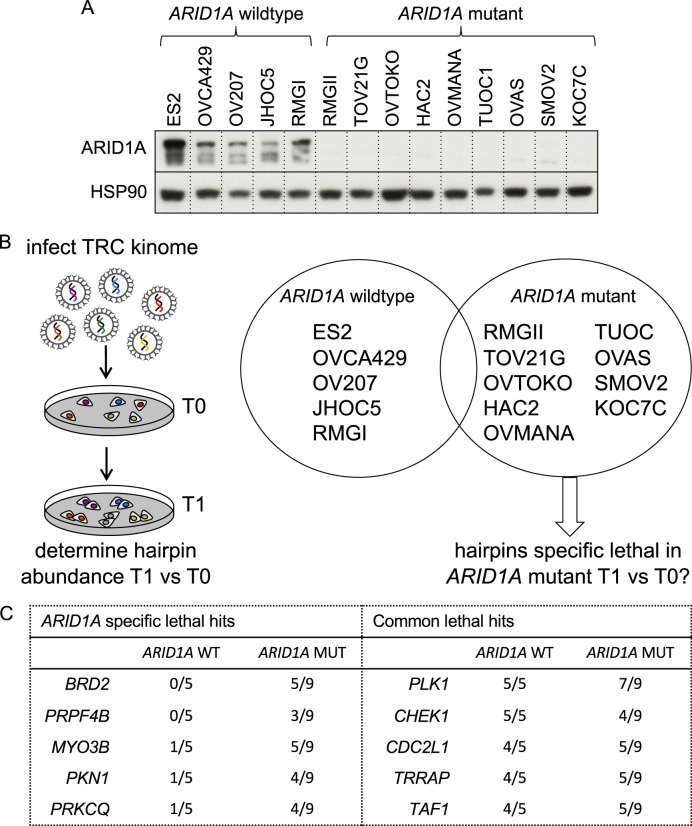
TRC kinome screen in OCCC cell panel. **a** ARID1A protein expression levels of the OCCC panel were measured by Western blot analysis. HSP90 was used as a loading control. **b** Each cell line from the OCCC panel was infected in triplicate with the lentiviral TRC kinome library with a multiplicity of infection below 0.5 and with cell amounts ensuring 1000× coverage of the library. Upon stable selection of the library timepoint zero (*T*0) was collected, followed by culturing of the cells for an additional two weeks, after which timepoint one (*T*1) was harvested. The relative abundance of the hairpins in *T*0 versus *T*1 was determined by deep sequencing. **c** Ranked lists for the lethal kinases were generated for every OCCC line based on three criteria (fold depletion, Geometric mean value of all hairpins and Second-best gene rank according to RIGER) as outlined in the methods section. From these ranked hit lists we determined which genes were lethal upon knockdown in all cell lines (common lethal hits) or preferentially in the *ARID1A* mutant lines (*ARID1A* specific lethal hits) and a top 5 is shown

**Fig. 2:**
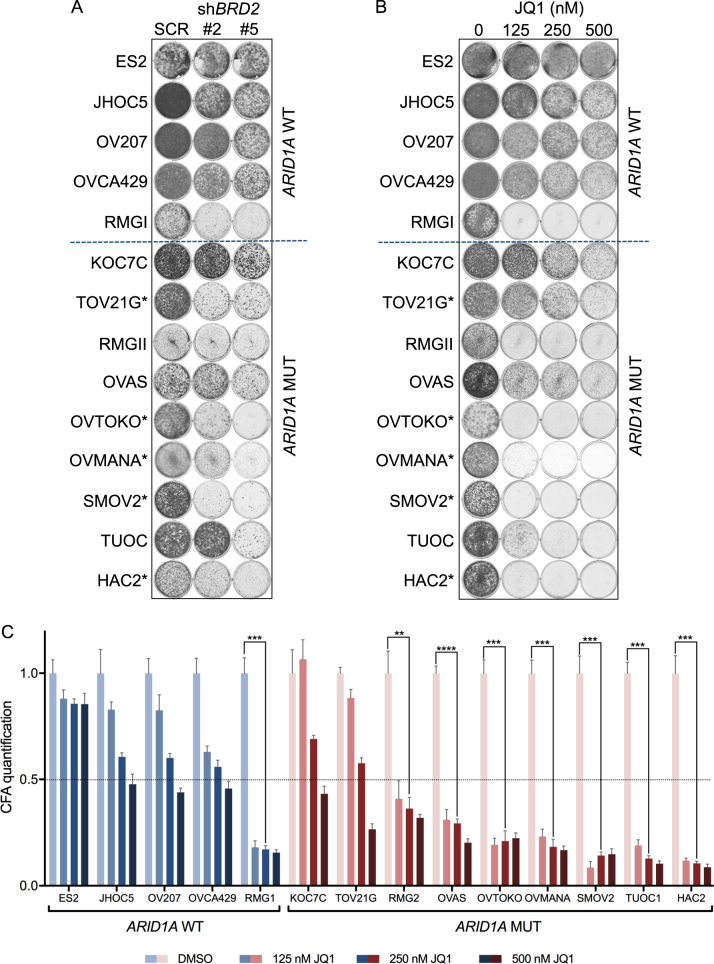
BRD2 validates as *ARID1A* mutant specific lethal hit. **a** The functional phenotypes of non-overlapping lentiviral sh*BRD2* vectors (#2 and #5) in the OCCC cell line panel were measured in a long-term colony formation assay. Cells expressing a mixture of nonfunctional scrambled hairpins (SCR) were used as control. The cells were fixed, stained and photographed after 10–12 days. In the cell lines labeled with an asterisk (*) *BRD2* was identified as lethal hit. **b** The indicated *ARID1A* wildtype and mutant OCCC cell lines were plated in 6-well plates (10,000 cells per well) and exposed to increasing JQ1 (0, 125, 250, and 500 nM) concentrations in triplicate. The cells were fixed, stained with crystal violet and photographed after 10 days. **c** Colony formation assays (CFA) from **b** were quantified by crystal violet dye extraction. Shown is relative reduction in crystal violet staining compared to untreated control. Colony formation assays were performed in triplicate. Error bars denote SD. The dotted line denotes the cut-off used (below 0.5 at 250 nM JQ1 concentration) to qualify cell lines as “JQ1 sensitive”. A Fisher Exact test with the abovementioned cutoff at 250 nM JQ1 gave the statistic value of 0.09. *P*-values (only shown for the “JQ1 sensitive” lines) were calculated with multiple *t*-tests, asterisk denote the number of digits after the decimal

To investigate which vulnerabilities exist in *ARID1A* mutant OCCC lines we collected a very sizeable and unique set of OCCC cell lines and validated their ARID1A status by both *ARID1A* sequencing and ARID1A protein expression analysis. We screened in total 14 OCCC cell lines (5 *ARID1A* wildtype and 9 *ARID1A* mutant, Fig. 1a) with a lentiviral shRNA library covering the human kinome to search for kinases whose suppression is specifically lethal in an *ARID1A* mutant context. We have previously described kinome-centered screening approaches using this library [7, 8]. In short, each cell line was infected with the kinome shRNA library in triplicate, selected for presence of viral integration, cells were harvested at timepoint zero (*T*0) and the remainder was replated and cultured. After two to three weeks, cells were collected for timepoint one (*T*1). Genomic DNA was isolated from both populations, and the relative abundance of the short hairpin sequences was determined by deep sequencing (Fig. 1b). For the selection of synthetic lethal genes, we set several criteria to ensure identification of hits with significant toxicity and with multiple hairpins (see Methods). Following these criteria, we generated ranked lists for the lethal kinases per cell line (Figure S1 and S2). By analyzing these cell-specific ranked lists we could identify common lethal kinases (*PLK1*, *CHEK1*, *CDC2L1*, *TRRAP*, and *TAF1*) and kinases that were more often lethal in the *ARID1A* mutant cell lines (*BRD2*, *PRPF4B*, *MYO3B*, *PKN1*, and *PRKCQ*) (Fig. 1c). The lethal kinases that were exclusively selected in the *ARID1A* mutant lines (*BRD2* and *PRPF4B)* were further validated. We subsequently observed that *PRP4FB* loss was lethal in all the OCCC cell lines tested (data not shown), so toxicity appeared independent of *ARID1A* mutation status. Interestingly, *BRD2* depletion using two independent shRNA sequences (validated on protein levels Figure S3A) recapitulated the screening data more convincingly (Fig. 2a, Figure S3B). Especially in those *ARID1A* mutant lines where *BRD2* was previously identified as lethal hit (TOV21G, OVTOKO, HAC2, SMOV2, and OVMANA), significant killing with two sh*BRD2* constructs was observed (with one sh*BRD2* resulting in at least 70% cell number reduction) and *BRD2* was therefore considered as the only hit from our ARID1A synthetic-lethal screens. Validation of BRD2 depletion in the complete OCCC panel identified the cell line RMGI as one notable outlier, as *BRD2* knockdown appeared to be toxic in this cell line whereas BRD2 was not identified as a lethal hit in the RMGI screen. Possibly, during validation *BRD2* shRNAs were introduced at higher titer compared to screening conditions leading to better knockdown and toxicity during validation procedure. Of note, we identified comparable BRD2 and BRD4 protein and RNA levels across the OCCC cell line panel indicating that different sensitivities towards *BRD2* knockdown cannot be explained by the BRD2 and BRD4 status of the individual cell line (Figure S4A, B, C).

### BET domain inhibition specifically inhibits the proliferation of *ARID1A* mutant OCCC lines

To further validate whether *BRD2* depletion caused proliferation defects predominantly in an *ARID1A* mutant context, we targeted BRD2 function with the use of BET inhibitors. Selective inhibitors targeting BET proteins BRD2, BRD3, BRD4 as well as BRDT, have been described to exhibit antineoplastic activity [9]. BET inhibitors are currently in clinical trials for various hematological malignancies and solid tumors. BET inhibitors bind to acetylated lysine recognition motifs on the thereby preventing binding of BET proteins to the chromatin, resulting in disruption of subsequent chromatin remodeling and gene expression [10]. First, we tested the BET inhibitor JQ1 on our OCCC panel using a long-term proliferation assay. Interestingly, JQ1 sensitivity closely matched the *BRD2* knockdown lethality across the OCCC cell line panel (Fig. 2a, b). Thus, *ARID1A* mutant cells appear more sensitive to BET inhibition than *ARID1A* wildtype cell lines. From the JQ1 colony formation assays we conclude that, based on a cutoff of 50% growth reduction at 250 nM JQ1, 1 out of 5 *ARID1A* wildtype cell lines and 7 out of 9 *ARID1A* mutant cell lines are sensitive to the BET inhibitor (Fig. 2c). Collectively, these findings demonstrate that *BRD2* knockdown lethality closely resembles JQ1 sensitivity in OCCC lines, and that *ARID1A* mutant lines are most sensitive to BET inhibition.

### *ARID1A* depletion enhances sensitivity to JQ1

**Fig. 3:**
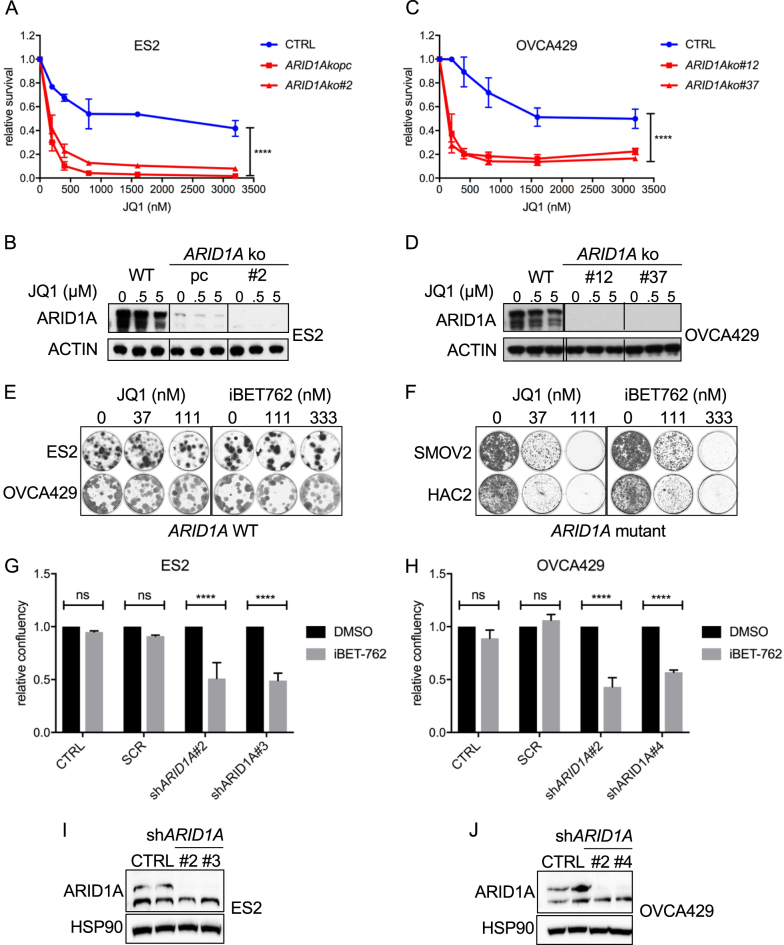
*ARID1A* loss induces sensitivity towards BET inhibitors. **a**, **c** ES2 and OVCA429 *ARID1A* knockout cell lines were generated with a dual vector doxycycline inducible CRISPR/Cas9 vector system. For ES2, wildtype (wt) cells, one polyclonal knockout line (*ARID1A* kopc) and one monoclonal knockout line (*ARID1A* ko#2) and for OVCA429, wt cells and two monoclonal knockout lines (*ARID1A* ko#12 and #37) were tested for JQ1 sensitivity in a 384 well 6-day cell viability assay using CellTiter Blue (CTB) as a readout. CTB measurements were normalized to untreated controls. Error bars denote SD. *P*-values were calculated with 2-way ANOVA, asterisk denote the number of digits after the decimal. **b**, **d** ARID1A Western blot analysis of ARID1A protein levels in the ES2 polyclonal *ARID1A* knockout clone and ES2/OVCA429 monoclonal *ARID1A* knockout clones. **e**, **f** The indicated two *ARID1A* wildtype and *ARID1A* mutant lines were exposed to increasing doses of BET inhibitors JQ1 and iBET762. After 10–12 days cells were stained and photographed. **g**, **h** ES2 and OVCA429 cell lines were stably infected with the indicated shRNA constructs targeting *ARID1A* and plated in 384 well plates. Confluency was monitored in the absence and presence of the BET inhibitor iBET762 (800 nM). Shown are Incucyte confluency measurements relative to untreated cells from three independent experiments. Error bars denote SD. *P*-values were calculated with multiple t-tests, asterisk denote the number of digits after the decimal. **i**, **j** Western blots were performed on the ES2 and OVCA429 cell lines stably infected with the indicated sh*ARID1A* constructs to monitor efficiency of *ARID1A* knockdown. HSP90 served as a loading control

The genetic heterogeneity of the OCCC cell line panel poses a possible limitation in determining a genotype-specific toxicity. To firmly establish *ARID1A* mutation as a direct cause of enhanced BET inhibitor sensitivity, we chose to test our findings further in OCCC isogenic cell line pairs. For this, we generated *ARID1A* knockout subclones from the *ARID1A* wildtype cell lines ES2 and OVCA429 using CRISPR/Cas9 targeting. In both a polyclonal ES2 cell line having significant ARID1A reduction as well as a full knockout ES2 single cell clone, we observed that loss of *ARID1A* significantly sensitized to BET inhibition by JQ1 (Fig. 3a, b). Similarly, the *ARID1A* knockout OVCA429 subclones acquired enhanced sensitivity to JQ1 inhibition (Fig. 3c, d). These findings demonstrate a direct causal link between loss of ARID1A function and sensitivity towards BET inhibitors. We noted that the *ARID1A* knockout clones adapted upon prolonged culturing (within months) in such a way that they gradually lost their enhanced sensitivity towards JQ1 inhibition (data not shown). This gradual adaptation to a drug-tolerant state has been observed before in other cell models [11]. Although speculative, we hypothesize that tumor-derived *ARID1A* mutant OCCC lines may have acquired additional genetic alterations causing a more stable phenotype compared to cell lines generated by CRISPR/Cas9 mediated *ARID1A* manipulation.

### JQ1 and iBET762 have similar activity in *ARID1A* deficient OCCC lines

To further validate our findings, we tested another BET inhibitor, iBET762. First, we compared the inhibitors JQ1 and iBET762 in a long-term proliferation assay in two *ARID1A* wildtype (ES2 and OVCA429) and two *ARID1A* mutant (SMOV2 and HAC2) OCCC lines. The *ARID1A* mutation dependent toxicity of BET inhibition appeared extremely similar for JQ1 and iBET762 in these cell lines (Fig. 3e, f). Next, iBET762 was tested on both ES2 (Fig. 3g) and OVCA429 (Fig. 3h) wildtype and *ARID1A* knockdown clones. Knockdown efficiencies for two independent sh*ARID1A* constructs were checked at the protein level (Fig. 3i, j). Upon *ARID1A* knockdown, both OCCC lines displayed increased iBET762 lethality in these experiments (Fig. 3g, h). Collectively, we conclude that BET inhibition by the addition of either JQ1 or iBET762 is more toxic in *ARID1A* mutant lines, further validating our *BRD2* screen hit.

### BET inhibition directly downregulates ARID1B expression

**Fig. 4:**
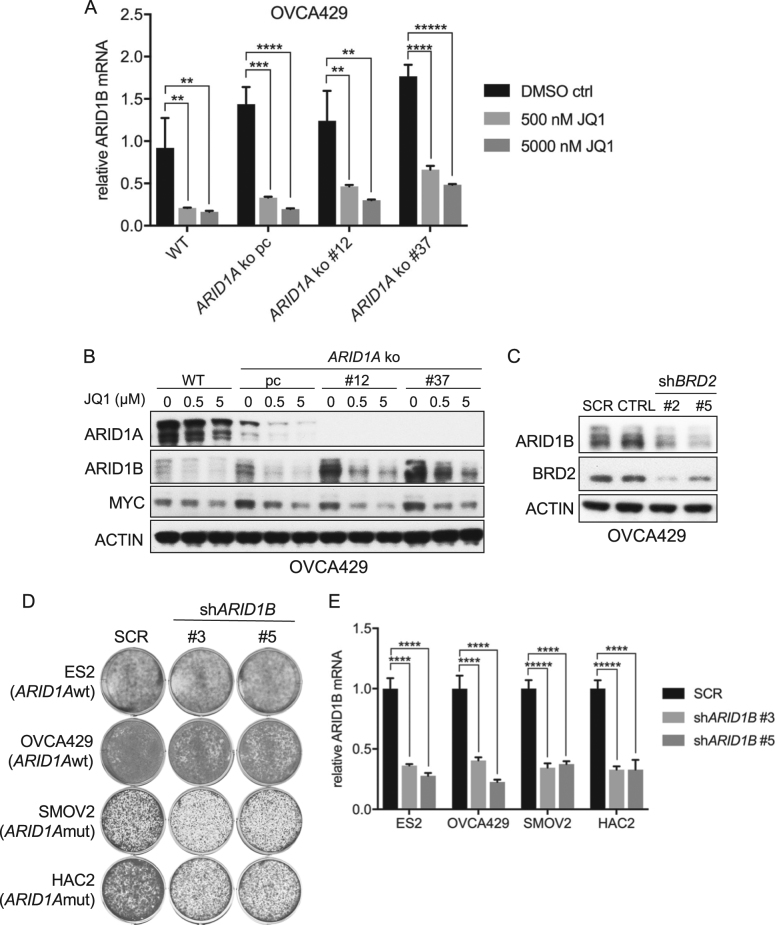
BET inhibition reduces ARID1B levels. **a** OVCA429 *ARID1A* knockout cell lines were generated with a dual vector doxycycline inducible CRISPR/Cas9 vector system. Wildtype cells, one polyclonal knockout line (*ARID1A* kopc) and two monoclonal knockout lines (*ARID1A* ko#12 and #37) were exposed to increasing amounts of JQ1 as indicated. After 48 h drug exposure *ARID1B* RNA analysis was performed. mRNA levels were normalized to expression of GAPDH. Error bars denote SD. *P*-values were calculated with multiple *t*-tests, asterisk denote the number of digits after the decimal. **b** The OVCA429 cells described in **a** were subjected to Western blot analysis for the indicated proteins. ACTIN served as a loading control. **c** Western blot analysis of ARID1B and BRD2 in OVCA429 cells stably infected with lentiviral shRNA vectors targeting *BRD2*. ACTIN served as a loading control. **d** Doxycycline inducible lentiviral *ARID1B* shRNA vectors #3 and #5 were introduced in two *ARID1A* wildtype and two *ARID1A* mutant OCCC lines. After stable selection cells were plated in a long-term proliferation assay in the presence of doxycycline. Cells expressing a mixture of nonfunctional scrambled hairpins (SCR) were used as control. The cells were fixed, stained and photographed after 10–14 days. **e** *ARID1B* mRNA expression analysis by qRT-PCR in ES2, OVCA429, SMOV2 and HAC2 cells stably expressing the two doxycycline-inducible sh*ARID1B* vectors. mRNA levels were normalized to expression of GAPDH. Error bars denote SD. *P*-values were calculated with multiple *t*-tests, asterisk denote the number of digits after the decimal

We next sought to investigate why *ARID1A* deficient cells exhibit an enhanced sensitivity towards BET inhibition. BET inhibitors likely exert a broad effect on transcription. A recent study identified *ARID1B*, a SWI/SNF member mutually exclusive with *ARID1A* in this complex, as a gene critically required for the survival of *ARID1A* mutant cell lines [12]. Based on this we tested whether BET inhibition had an impact on ARID1B expression. First, we tested the effect of increasing amounts of JQ1 on *ARID1B* expression in the OVCA429 *ARID1A* wildtype and knockout cell lines. The differential sensitivity of these isogenic lines towards JQ1 was already demonstrated in Fig. 3. We indeed observed a concentration-dependent downregulation of *ARID1B* expression both at RNA (Fig. 4a) and protein levels (Fig. 4b). MYC protein levels were included as a positive control for effective inhibition. Similar results were obtained in the ES2 *ARID1A* wildtype and knockout cell lines (data not shown). Second, we observed that *BRD2* knockdown resulted in reduced ARID1B protein levels, directly implicating BRD2 in the regulation of *ARID1B* expression (Fig. 4c). Moreover, after knockdown of *ARID1B* expression in several OCCC lines, we observed toxicity only in an *ARID1A* mutant background, in agreement with earlier published experiments (Fig. 4d, e). Of note, when testing the *ARID1A* mutant lines in which *BRD2* was not identified as a hit (KOC7C, OVAS, RMGII and TUOC1), we observed significant toxicity of *ARID1B* knockdown in those lines that also displayed significant BETi toxicity (OVAS, RMGII, and TUOC1), suggesting that *BRD2* was probably not identified as a hit due to our hit-selection criteria or technical variations during screening procedures (Figure S4D,E). We noted that exogenous *ARID1B* overexpression could not rescue JQ1 mediated growth inhibition (data not shown) suggesting that multiple factors may be involved in JQ1 mediated cell toxicity. In accordance with previous studies [13], we noticed significant toxicity upon *ARID1B* overexpression, which complicated these rescue experiments considerably. Collectively, our findings suggest that down-modulation of *ARID1B* by BET inhibitors contribute to the *ARID1A* mutant specific vulnerability in OCCC cell lines, but likely is not solely responsible for the observed growth defects.

### BET domain inhibition inhibits expression of other SWI/SNF members

**Fig. 5:**
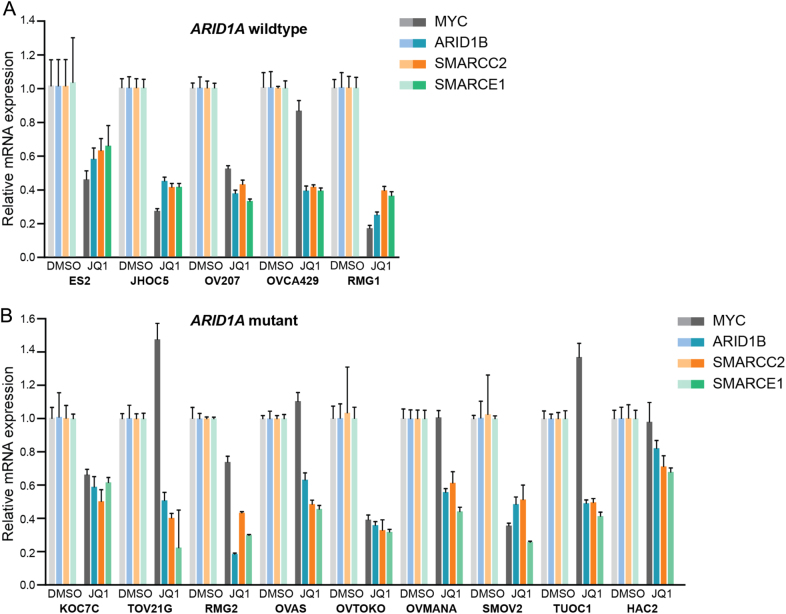
BET inhibitors target multiple SWI/SNF members. **a**, **b** ARID1A wildtype (**a**) and ARID1A mutant (**b**) OCCC lines were exposed for 40 h to 500 nM JQ1 and were subjected to mRNA expression analysis with qRT-PCR. mRNA levels were normalized to expression of GAPDH and displayed is the relative expression of the indicated mRNAs to untreated cells. Error bars denote SD

When analyzing RNAseq data from OVCA429 *ARID1A* wildtype and knockout cells exposed to JQ1, we observed that BET inhibition resulted in transcriptional repression of several SWI/SNF family members besides *ARID1B*, including *SMARCC2* and *SMARCE1* (data not shown). As such, BET inhibition may interfere with SWI/SNF function by affecting the transcription of multiple components of this multi-protein complex. To examine the generality of this observation we tested the effect of JQ1 exposure on gene expression of the SWI/SNF members *ARID1B*, *SMARCC2* and *SMARCE1* in the complete OCCC panel. *MYC* was taken along as a control. Indeed, JQ1 treatment of the complete OCCC cell line panel resulted in a robust downregulation of the indicated SWI/SNF RNA levels, demonstrating a general effect of BET inhibition on the gene expression of SWI/SNF members (Fig. 5a, b). Notably, *MYC* RNA levels were downregulated by JQ1 exposure only in a subset of OCCC cell lines, suggesting that *MYC* repression by bromodomain inhibition is not a general phenomenon in OCCC cell lines.

**Fig. 6:**
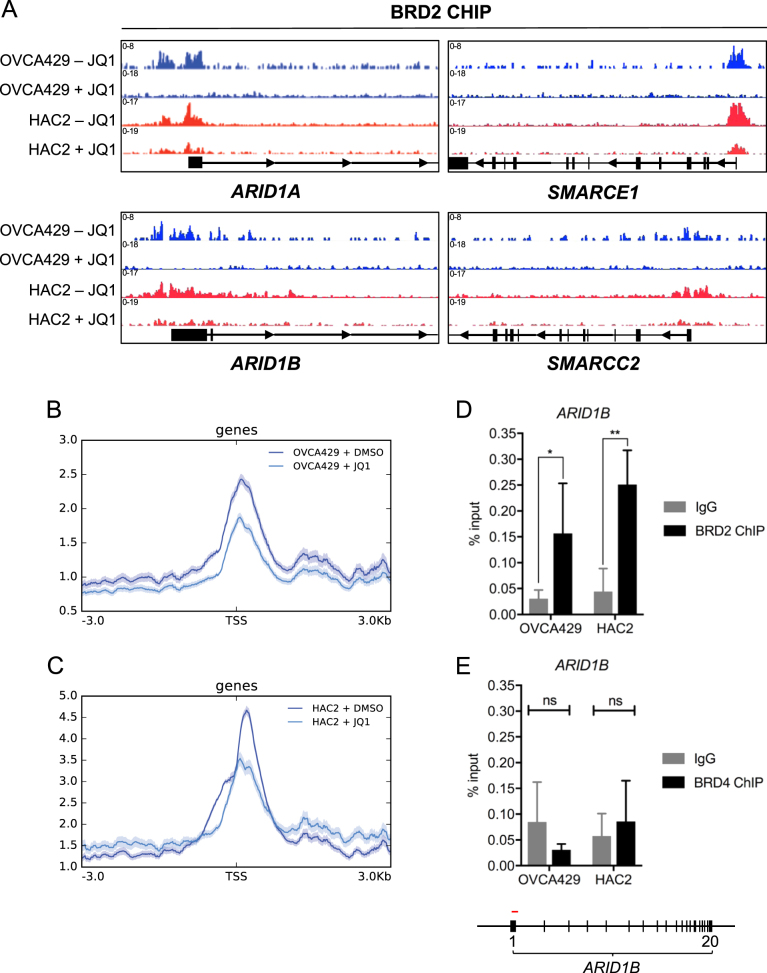
Direct binding of BRD2 in *ARID1B* locus. **a** The *ARID1A* wildtype line OVCA429 and the *ARID1A* mutant line HAC2 were cultured for 24 h in 500 nM JQ1 or DMSO vehicle control and subjected to chromatin immunoprecipitation with BRD2 and control antibodies. ChIP sequences were generated by Illumina Hiseq 2000 genome analyzer and aligned to the Human Reference Genome (assembly hg38) and visualized in IGV. Displayed are IGV snapshots of the Transcriptional Start Sites (TSS) of the indicated SWI/SNF members *ARID1A*, *ARID1B*, *SMARCE1* and *SMARCC2*. **b**, **c** Displayed are the average profiles of ChIP-seq signal for the indicated cell lines in absence and presence of JQ1 at transcriptional start site regions (+/−3 kb) (*n* = 66035). Shading indicates standard error of average read count profiles. **d**, **e** qRT-PCR amplification was performed with primers located in the *ARID1B* gene. Shown is the percentage of the BRD2 (**d**) and BRD4 (**e**) and control IgG chromatin immunoprecipitations over chromatin input qRT-PCR amplification. Error bars denote SD over *n* = 4 (**d**) and *n* = 3 (**e**) experiments. *P*-values were calculated with multiple t-tests, asterisk denote the number of digits after the decimal. Primer location is visualized in red in the gene map displayed underneath the graph

Interestingly, chromatin immunoprecipitation experiments demonstrated specific BRD2 binding to various SWI/SNF member promoter regions, including *ARID1A*, *ARID1B*, *SMARCE1*, and *SMARCC2*, both in *ARID1A* wildtype (OVCA429) and *ARID1A* mutant (HAC2) cell lines (Fig. 6a). Furthermore, JQ1 treatment efficiently inhibited BRD2 chromatin binding in OVCA429 and HAC2 (Fig. 6b, c, Figure S5). The BRD2 ChIP sequencing data were verified by qPCR of the *ARID1B* promoter region (Fig. 6d). Of note, in this experiment we also included a BRD4 ChIP and observed no binding of BRD4 to the same *ARID1B* promoter region (Fig. 6e). These observations suggest that the effects of BRD2 inhibition on *ARID1B* expression and other SWI/SNF members are specific and direct.

### In vivo OCCC models demonstrate *ARID1A* dependent sensitivity to JQ1

**Fig. 7:**
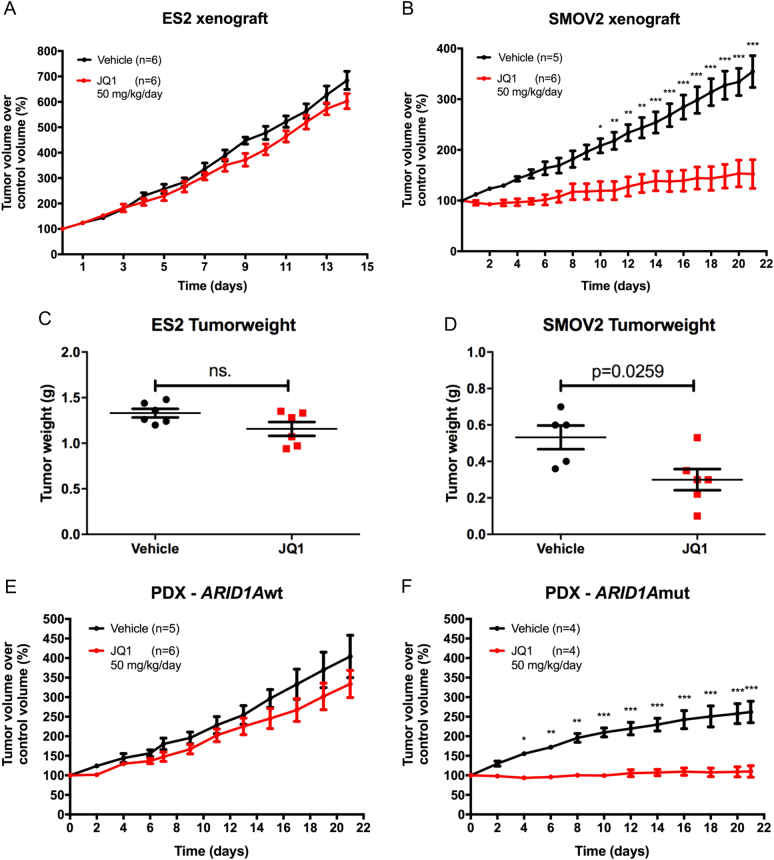
JQ1 specifically inhibits in vivo growth of *ARID1A* mutant OCCC xenografts and PDX models. **a**, **b** *ARID1A* wildtype ES2 cells (5 × 10^6^ in PBS) were subcutaneously injected in the flank of 8–10-weeks-old NSG mice. For the *ARID1A* mutant SMOV2 xenograft experiments, successfully established SMOV2 engraftments (see methods) were dissected, and tumor pieces were subcutaneously propagated in the flank of new mice. When tumors reached ~200 mm^3^, mice were randomized into vehicle (DMSO) control or treatment (JQ1) groups (*n* = 6 mice/group). ES2 xenografts reached the 1500 mm^3^ endpoint after 14 days of JQ1 administration, SMOV2 xenografts were treated with JQ1 for 21 days. **c**, **d** Displayed are the tumorweights of the indicated excised tumors after vehicle or JQ1 treatments. **e**, **f** *ARID1A* wildtype (**e**) and mutant (**f**) PDX F3 tumors were established (see methods) and tumor pieces were subcutaneously implanted in the flank of NSG mice. When tumors demonstrated sustained growth, mice were randomized into vehicle (DMSO) control or treatment (JQ1) groups. Treatment with JQ1 was continued for 21 days. For all in vivo experiments, JQ1 (50 mg/kg) or DMSO vehicle were administered intraperitoneally daily and tumor growth was quantified by caliper measurements. Tumor growth was determined as tumor volume treatment day/ tumor volume start treatment. Statistical significance for tumor growth was determined using two-way ANOVA with Bonferoni post-hoc test correction. Error bars denote standard error of mean

To test the *ARID1A* dependent sensitivity towards BET inhibition in vivo, we used NSG mice xenografted with OCCC cell lines. Unfortunately, engraftment efficiency of OCCC cells appeared rather low since from the tested ES2, SMOV2, TUOC1, and HAC2 cell lines, only ES2 and SMOV2 grew in mice. For the *ARID1A* wildtype ES2 xenograft cohorts, we did not observe a significant growth difference between the vehicle and JQ1 treated tumors (Fig. 7a). In contrast, the *ARID1A* mutant SMOV2 xenografts were significantly growth impaired upon JQ1 treatment (Fig. 7b). In agreement with this, the tumor weights of excised tumors were not significantly reduced in JQ1 treated ES2 xenografts (Fig. 7c) but significantly declined in the JQ1 treated SMOV2 xenografts (Fig. 7d). Furthermore, Western blot analysis of SMOV2 tumor lysates demonstrated a significant reduction of ARID1B expression upon JQ1 treatment compared to DMSO vehicle control (Figure S6A). Thus, upon prolonged exposure in an in vivo model, BET inhibition downregulates ARID1B, likely contributing to the enhanced sensitivity of these tumor xenografts to JQ1. Given the low engraftment efficiencies of the OCCC cell lines, we reasoned that OCCC PDX models could provide an alternative strategy to further validate our results in vivo. F3 tumor pieces from an *ARID1A* wildtype and an *ARID1A* mutant (homozygous *ARID1A* 1148* stop-gained mutation) PDX model (Figure S6B), were subcutaneously implanted in NSG mice, and were randomized into vehicle control and JQ1 treatment groups. For the PDX-*ARID1A*-wildtype cohort, JQ1 treatment did not significantly impair tumor growth compared to vehicle control treatment (Fig. 7e). Importantly, tumor growth in the PDX-*ARID1A*-mutant cohort was greatly impaired by JQ1 treatment (Fig. 7f). Thus, in agreement with the OCCC cell line xenograft experiments, *ARID1A* mutant PDX tumors are sensitive to JQ1, whereas *ARID1A* wildtype PDX tumors are unaffected by JQ1 treatment. Of note, Ki67 staining was stronger reduced upon JQ1 treatment in the *ARID1A* mutant PDX tumors, whereas Cleaved Caspase3 staining of the JQ1 treated tumors revealed a small but significant increase in apoptotic cells in the *ARID1A* mutant PDX tumors (Figure S6C, D). Collectively, these observations support the notion that BET inhibitors are potentially useful for the treatment of *ARID1A* mutant OCCC.

## Discussion

Through loss of function genetic screens in a large panel of OCCC cell lines, we identify *BRD2* loss as an *ARID1A* mutation-specific dependency in OCCC cell lines. BRD2 is a member of the BET family of proteins consisting of BRD2, BRD3, BRD4, and BRDT. Bromodomain proteins are epigenetic readers, that play a role in transcriptional regulation through binding of hyperacetylated chromatin. BET proteins have also been reported to act as protein kinases, explaining their presence in the kinome library [14]. Importantly, inhibiting BRD2 function resulted in enhanced sensitivity of the *ARID1A* mutant OCCC lines. Of note, *BRD4* loss appeared to be toxic in various OCCC lines independent of *ARID1A* status, whereas *BRD3* and *BRDT* were not selected as lethal hits in our screens (Figs. S1,2). Presumably, the BET family members exert diverse and only partially overlapping functions in OCCC cell lines causing only loss of the BET member *BRD2* to be synthetic lethal with *ARID1A* mutation. In line with this assumption, a recent study reported a very different genome-wide occupancy of BRD2 and BRD4, suggesting non-redundant genomic functions [15]. Our observation that specifically BRD2, and not BRD4, binds to the *ARID1B* promoter region suggests that BRD2 knockdown may have different effects on residual SWI/SNF function in *ARID1A* mutant OCCC lines than *BRD4* knockdown. Furthermore, gene essentiality data generated for luminal breast cancer cell lines recently uncovered a BET-independent requirement for BRD4, demonstrating that *BRD4* knockdown lethality not always coincides with JQ1 sensitivity [16]. Indeed, also in our OCCC cell lines *BRD4* knockdown lethality could not predict JQ1 response. However, more detailed information on the inhibitory efficiencies of both JQ1 and iBET762 on the different bromodomain family members in OCCC lines will be required to support this hypothesis. Interestingly, we show that *ARID1A* depletion directly sensitized OCCC lines to BET inhibition, further strengthening the notion that *ARID1A* loss is critical to the observed phenotype. Currently, we have no indications that *ARID1A* loss sensitizes other cancer types to BET inhibition, suggesting an OCCC-specific context dependency for the findings we report here.

Our data suggest that *ARID1B* transcriptional down-modulation by BET inhibition contributes to the *ARID1A* mutant dependent toxicity. However, we cannot rule out that other (SWI/SNF) factors regulated by members of the BET domain family contribute to the observed phenotypes. It has been stated previously that ARID1B may be a potential therapeutic target for *ARID1A* mutant cancers [12]. Importantly, we here demonstrate that BET inhibition may provide a way to target ARID1B indirectly, which could be explored further therapeutically. In that light, it is encouraging that the *ARID1A* dependent sensitivity to BET inhibitors was also observed in OCCC xenografted mice treated with the BET inhibitor JQ1. Unfortunately, more extensive in vivo cell line validation experiments were complicated by the low tumor-take rate of xenografted OCCC cell lines. Therefore, the OCCC PDX experiments reported here provide important additional evidence that BET inhibition imposes an *ARID1A* mutant dependent toxicity in an in vivo model.

It has been reported that inhibition of the methyltransferase EZH2 may represent a novel treatment strategy for *ARID1A* mutant cancers [17]. Furthermore, recent studies have demonstrated a specific sensitivity of *ARID1A* mutant OCCC cells to either dasatinib [18] or to the HDAC6 inhibitor ACY1215 [19]. For future studies it will be of interest to test these different synthetic lethal interactions together with our present findings to compare their ability to specifically target *ARID1A* mutant OCCC in vivo. Of note, the OCCC cell line panel used in our study to search for *ARID1A* mutation dependency is the largest reported to date, thereby most closely reflecting the mutation spectrum (e.g., concurrent hotspot mutations in *PIK3CA, KRAS*) found in OCCC patients [20]. As might be expected from such a heterogeneous group of cell lines, there was not a perfect separation between *ARID1A* mutant and wildtype cell lines in terms of BET inhibitor response. However, we believe that the remarkable sensitivity of the majority of *ARID1A* mutant OCCC lines for the BET inhibitors warrants further (pre)-clinical exploration.

In summary, we report here an unexpected and new synthetic lethal interaction between *BRD2* loss and *ARID1A* mutation. We suggest that the inhibitory effects on residual SWI/SNF function, specifically via reduced *ARID1B* expression, may explain the enhanced sensitivity of *ARID1A* mutant cells to BET inhibitors. Our data imply that patients with *ARID1A* mutant OCCC may benefit from BET domain inhibitors added to their treatment regimen.

## Materials and methods

### OCCC cell lines and genotyping

TOV21G was obtained from ATCC; OVTOKO, RMGI, RMGII, OVMANA, HAC2 from JCRB Cell Bank; JHOC5 from RIKEN Cell Bank; OVCA429 from Cell Biolabs; TUOC1, OVAS, SMOV2, and KOC7C were kindly provided by Hiroaki Itamochi; ES2 was a kind gift from Els Berns and OV207 was a kind gift from Vijayalakshmi Shridhar. All cells were maintained in RPMI supplemented with 10% Fetal Calf Serum and 100 µg/ml Penicilin/Streptomycin and 2 mM l-Glutamine and tested negative for mycoplasma contamination. The Haloplex sequencing custom platform from Agilent was used to determine *ARID1A* mutation status, with the target region design based on NM_139135 and NM_006015. OCCC cells were classified as “*ARID1A* mutant” when homozygous frameshift and/ or nonsense *ARID1A* mutations were detected in combination with no detectable ARID1A protein on Western.

### TRC kinome screen

A kinome-centered short hairpin RNA (shRNA) library targeting 535 human kinases was assembled from the TRC human genome-wide shRNA collection (TRCHs1.0). Each OCCC line was stably transduced by lentiviral infection in triplicate with a multiplicity of infection (MOI) below 0.5 and sufficient number of cells to ensure a 1000-fold library coverage. A *T*0 time point sample was taken from the cells stably expressing the shRNA library and the remainder of the cells was cultured for 2–3 weeks after which *T*1 was harvested. The relative abundance of each shRNA comparing *T*1 to *T*0 was determined using the R/Bioconductor package DESeq [21]. Kinases were considered as lethal hits when the *P* value calculated using DESeq was lower than 0.1 and the following three criteria were fulfilled: (1) at least one hairpin with a Fold Change below 0.3 (to ensure significant toxicity), (2) a geometric mean of all the hairpins below 0.8 (to ensure that the majority of hairpins have a similar effect, reducing false-positive results), (3) a top 100 ranking according to the Second Best Gene Rank in RIGER (a method to rank genes by the rank of the second best scoring hairpin of that gene, ensuring selection of genes with at least two functional hairpins) [22]. The DESeq screening data for the 14 OCCC lines are enlisted in Supplementary Data file S1.

### Plasmids

The following TRC pLKO.1 shRNA vectors were used for validation: *BRD2*#2: TRCN0000006309; *BRD2*#5: TRCN0000006312. The lentiviral vector pLKO.1-Scramble shRNA was obtained from Addgene (#1864). A doxycycline inducible lentiviral shRNA vector (GINSENG) [23] was modified as described [24] and was used to express the *ARID1B* hairpins (#3: GGAAGATTAGAGGGTCACATA and #5: GCCGAATTACAAACGCCATAT) under the control of doxycycline. *ARID1A* knockout cell lines were generated with a dual vector doxycycline inducible CRISPR/Cas9 vector system (iKRUNC) as described [25] using the gRNA sequence targeting *ARID1A*: AGGATGAGTCACGCCTCCAT. pLKO was used to express the *ARID1A* shRNA hairpins with RNAi target sequences *ARID1A*#2: AGTTGAAGTTCTGATGAA, *ARID1A*#3: GAGAAGTTGTATAGCACTA and *ARID1A*#4: GTGTAGACCCTTTCATGTA.

### Antibodies and compounds

For Western blotting primary antibodies against ARID1A (PSG3), ACTIN (C2), HSP90 (H-114), CMYC (N-262), BRD4 (H-250) were obtained from Santa Cruz Biotechnology; BRD2 (A302-582A) from Bethyl; ARID1B (AB57461) and Histone-H3(trimethylK27) (AB6002) from Abcam. Immunohistochemical analysis of paraffin-embedded xenograft slices were performed as described [26], using antibodies against Cleaved-Caspase3 from Cell Signaling (#9661); Ki67 from DAKO (M7240) and ARID1A from Sigma (HPA005456). iBET762 was obtained from Xcess Biosciences. JQ1 for the cell line experiments was kindly provided by the Bradner lab (Dana-Farber Cancer Institute, Boston, USA) [9] and later purchased from Axon Medchem (axon 1989); both batches had similar activity. JQ1 (HY-13030) used in the xenograft experiments was purchased from MedChem Express. Before use in animals, the in vitro activity of JQ1 (HY-13030) was compared to the activity of JQ1 obtained from Axon Medchem.

### Quantitative RT-PCR

The 7500 Fast Real-Time PCR System from Applied Biosystems was used to measure mRNA levels which were normalized to expression of *GAPDH*. Each QRT-PCR experiment included technical replicates and were repeated at least once. The following primer sequences were used in the SYBR® Green master mix (Roche): *GAPDH*_Forward, AAGGTGAAGGTCGGAGTCAA;

*GAPDH*_Reverse, AATGAAGGGGTCATTGATGG;

*BRD2*_Forward, GAGGTGTCCAATCCCAAAAAGC;

*BRD2*_Reverse, ATGCGAACTGATGTTTCCACA;

*BRD4_*Forward, AATGAGCTACCCACAGAAGAAAC;

*BRD4_*Reverse, GAGTCGATGCTTGAGTTGTGTT;

*ARID1B*_Forward, CAAGGGGATCAGAGCAACCC;

*ARID1B*_Reverse, CTACCTGGGATACTTGCAGGA;

*SMARCC2*_Forward, TACTCTTGGGGGTTCAGTCG;

*SMARCC2*_Reverse, TCTTCAACGGCAAGAACAAG;

*SMARCE1*_Forward, AACAACTACAGGCTGGGAGG;

*SMARCE1*_Reverse, CGGCTTATCTGGTGGCTTT.

### Proliferation assays

For long-term colony formation assays, cells were cultured in 6-well plates and medium was refreshed every 3 days. After 10 days cells were fixed with 4% formaldehyde and stained with 0.1% crystal violet and subsequently scanned. Colony formation assay quantification was performed by optical density measurements of extracted dye at 590 nM. Proliferation assays were repeated at least three times, within one assay three technical replicates were performed. Representative stainings and quantifications are shown. Six-day growth assays were performed in quadruple in 384-well plates and were quantified with the cell viability assay CellTiter-Blue (Promega) or by confluency monitoring in an IncuCyte Zoom live cell imaging system (Essen BioScience). Measurements were normalized to untreated controls.

### Chromatin immunoprecipitation

Chromatin Immunoprecipitation (ChIP) was performed as described [27]. For each ChIP 4 µg anti-BRD2 (A302-583A) or anti-BRD4 (A301-985A) from Bethyl Laboratories and control Rabbit IgG SC-2027 from Santa Cruz Biotechnology were used. ChIP sequences were generated with the use of the Illumina Hiseq 2000 genome analyzer. Mapped reads were visualized in heatmaps and profiles using deepTools2 v2.4.0 with the UCSC hg38 refGene coordinates. QPCR of the *ARID1B* region was performed with ARID1B1.1_Forward, CGCCCACAATGTGCTTTAACGG; ARID1B1.1_Reverse, AGGAAAAACCCACTCGCTTGTC.

### OCCC xenografts and PDX models

All animal experiments were approved by the Institutional Animal Care and Use Committee of the University of Groningen and carried out in accordance with the approved guidelines. For the xenografts, ES2 cells (5 × 10^6^ in PBS) and SMOV2 cells (5 × 10^6^ in 50% PBS/50% Matrigel) were subcutaneously injected in the flank of 8 to 10 weeks old NOD.CB17-Prkdcscid/NCrHsd (NSG) mice. Due to the long latency time (on average 75 days), we used successfully established SMOV2 xenografts for subsequent experiments. For this, SMOV2 xenografts were dissected, and 3 × 3 × 3 mm^3^ pieces were subcutaneously propagated in the flank of new mice. STR profiling was used to confirm SMOV2 identity of established xenografts. Thus, ES2 cells were injected and SMOV2 tumor pieces were transplanted in the flanks of NSG mice, and as soon as tumors reached the threshold size of 200 mm^3^, mice were randomized into vehicle control or treatment groups (*n* = 6 mice/group). JQ1 (50 mg/kg in 10% DMSO, 9% (2hydroxypropyl)-β-cyclodextrin) or vehicle (10% DMSO, 9% (2hydroxypropyl)-β-cyclodextrin) was daily administered intraperitoneally. Treatment with JQ1 was continued for 21 days as described in previous studies [28, 29]. Based on the time to reach the humane endpoint for tumor size in mice (~1500 mm^3^), ES2 tumor-bearing mice had to be euthanized after 14 days of JQ1 treatment.

OCCC PDX models were established as described previously [26]. Briefly, all patients gave written informed consent and tumor specimens were obtained during surgery. Clinical characteristics of the patient from which *ARID1A* mutant OCCC PDX was established, were FIGO stage IIIC, no response to carboplatin/paclitaxel chemotherapy and a 9 months disease specific survival. *ARID1A* wildtype PDX was established from an OCCC patient FIGO stage IIB that showed a full response to carboplatin/paclitaxel chemotherapy and no relapse after 13 months. OCCC PDX models were sequenced using Haloplex (Agilent technologies) to determine *ARID1A* mutation status. F3 tumor pieces from an *ARID1A* wildtype and *ARID1A* mutant PDX model were cut into 3 × 3 × 3 mm^3^ pieces and subcutaneously implanted in NSG mice. When tumors demonstrated sustained growth (*ARID1A* wildtype PDX on average 26 days, *ARID1A* mutant PDX on average 34 days), mice were randomized into vehicle control or treatment groups (*n* = 5 mice/group). JQ1 (50 mg/kg in 10% DMSO, 9% (2hydroxypropyl)-β-cyclodextrin) or vehicle (10% DMSO, 9% (2hydroxypropyl)-β-cyclodextrin) was daily administered intraperitoneally. Treatment with JQ1 was continued for 21 days.

Sample size for mouse experiments were calculated to be four mice per group (using significance level alpha of 5%, power 80%, estimated effect in growth reduction 50% and coefficient variation of 25%). 1–2 additional mice per group were added in case of dropouts. Animals were excluded when no initial tumor growth was detected before treatment start or animals had >20% weight loss or died during treatment course. Variances between the groups being compared were similar.

## Electronic supplementary material

Figure S1. TRC kinome lethal hitlists for the ARID1A wildtype lines

Figure S2. TRC kinome lethal hitlists for the ARID1A mutant lines

Figure S3. BRD2 knockdown in the OCCC cell line panel

Figure S4. BRD2 and BRD4 status OCCC cell line panel

Figure S5. BRD2 ChIP-seq heatmaps at TSS for OVCA429 and HAC2

Figure S6. ARID1B protein analysis and H&E/ Cleaved-caspase3 staining of JQ1 treated tumors

Suppl Data file
