# Supplementary material for: ARID1A mutation sensitizes most ovarian clear cell carcinomas to BET inhibitors
Source: Oncogene. 2018 May 15;37(33):4611–25. doi: 10.1038/s41388-018-0300-6 (PMC6095834; doi:10.1038/s41388-018-0300-6)
Supplement: Supplementary file 1 — Figure S1. TRC kinome lethal hitlists for the ARID1A wildtype lines [file 41388_2018_300_MOESM1_ESM.pdf]

Suppl. Figure 1

ES2<br>*ARID1A* WT	RMGI<br>*ARID1A* WT	JHOC5<br>*ARID1A* WT	OVCA429<br>*ARID1A* WT	OV207<br>*ARID1A* WT
CDC2L1;CDC2L2	CDC2L1;CDC2L2	BUB1B	PTK2	CDC2L1;CDC2L2
PLK1;RPL37A	CHEK1	CDC2L1;CDC2L2	PRKAA1	CHEK1
BMPR1A	PLK1	SRPK3	TIE1	MAP4K2
DYRK1B	EGFR	PLK1;RPL37A	CHEK1	PAK1
CDKL4	POLD1	FGFR1	PLK1;RPL37A	PLK1
RIOK2	KDR	CHEK1	RIPK3	AURKB
CHEK1	PLK1;RPL37A	VRK2	AURKB	MAP4K3
TRRAP	ATR	ADRBK2	TRIM33	PLK1;RPL37A
BRD4	TAF1	RPS6KA4	ADRBK2	FRAP1
TGFBR1	VRK3	PTK7	MAP3K12	MYLK
SNF1LK2	SNF1LK2	ICK	CCL14;CCL15	SMG1
ALPK2	PI4KA	PIK3CB	EEF2K	ICK
ICK	TIE1	AURKB	WEE1	PRKAA1
TAF1	STK3	DDR1	EPHA5	TRRAP
EPHA5	PI4KB	MAPK6	PSKH1	MERTK
RPS6KL1	SMG1	TXK	MUSK	CDC2L6
PRKDC	VRK1	PRKAA1	MYO3B	BRSK2
CIT	PDPK1	KDR	PRKCQ	SNF1LK2
TIE1	PIK3R2	ABL2	SNF1LK2	PI4KB
BUB1B	BRD4	STK35	GRK6	AKT3
MYLK	ULK3	MYLK	OBSCN	DYRK1A
EEF2K	TRRAP	RAF1	SGK3	
SGK269		LONP1	DGKH	
NEK8		MAPK3	ITK	
EPHA6		TRIO	MYLK	
ABL1;BCR		NRBP2	CDKL3	
TLK1		ERBB3	TAF1	
PCTK1		ANTXR1	ERBB3	
RORA		PIK3C2G	PRKX	
RIPK3		PLK4	MARK1	
SPHK1		TAF1	CIT	
ADRBK2		FRAP1	MAP2K5	
ERBB3		RIPK3	FGFR1	
TRIB2		TNK2	ADCK5	
KSR2		SPHK1	RIPK2	
NR4A3		PTK2	PASK	
NPR1		TRRAP	PCTK1	
ALPK1		OBSCN	PTK7	
AURKA		STK16	TYK2	
ATM		ULK3	NTRK1	
CDKL1		EEF2K	ULK3	
ANTXR1		STK25	PFTK1	
JAK1		POLD1	FYN	
CDKL3		DGKH	PIK3R2	
CCL14;CCL15		BMPR1A	EPHA3	
FRAP1		ATR	MAP3K14	
SLTM		PCTK1	PKN1	
MAP3K7		EIF2AK1	MAST3	
NTRK2		PINK1	MLKL	
TSSK6		NTRK1	STK39	
PI4KB		CCL14;CCL15	INSRR	
PIK3R2		TEC	BMP2K	
BMP2K		TGFBR2	KSR1	
TYK2		PRKCZ	CLK2;CLK2P	
LMTK3		MARK1	RP6	
PIK3C2G		MELK	NEK6	
RIOK3		TYK2	RPS6KA4	
MMAB		SRPK2	PAK6	
DGKE		PDPK1	STRADA	
AMHR2		TRIM33	STK32A	
WEE1		CSNK1G2	SGK1	
MARK1		PRKDC	CSNK1G2	
MARK3		ATM;NPAT	NUAK1	
PIK3R1		SGK1	TEC	
ULK3		CLK2;CLK2P	SPEG	
PTK2		CIT	RAF1	
MAPK6		PASK	EPHA7	
KSR1		NPR1	PKLR	
SCYL1		KIAA1804	RORA	
AKT3		NEK7	NUAK2	
EIF2AK3		NUAK2	EPHB4	
KIAA0999		ERBB2	EPHA2	
CLASP1		RPS6KA3	RNMT	
PCTK2		PRKG1	PIK3R4	
TEX14		KSR2	TAOK2	
PRKG1		LYN		
DGKH		PIK3CD		
WNK1		MAP2K5		
DHDDS		EPHB4		
PTK7		CCL13		
HIPK4		NR4A3		
STK32A		MAP3K10		
CLK2;CLK2P				
ATR				
