# Supplementary material for: ARID1A mutation sensitizes most ovarian clear cell carcinomas to BET inhibitors
Source: Oncogene. 2018 May 15;37(33):4611–25. doi: 10.1038/s41388-018-0300-6 (PMC6095834; doi:10.1038/s41388-018-0300-6)
Supplement: Supplementary file 2 — Figure S2. TRC kinome lethal hitlists for the ARID1A mutant lines [file 41388_2018_300_MOESM2_ESM.pdf]

Suppl. Figure 2

TOV21G *ARID1A* mut	OVTOKO *ARID1A* mut	HAC2 *ARID1A* mut	OVMANA *ARID1A* mut	TUOC1 *ARID1A* mut	OVAS *ARID1A* mut	KOC7C *ARID1A* mut	RMGII *ARID1A* mut	SMOV2 *ARID1A* mut
SMG1	PLK1;RPL37A	WNK1	PLK1;RPL37A	CDC2L1;CDC2L2	SMG1	PRKAA1	MYO3B	CHEK1
CDC2L1;CDC2L2	EPHB2	ERBB4	CDC2L1;CDC2L2	PLK1;RPL37A	CDC2L1;CDC2L2	MYO3B	PIK3R2	MYO3B
PLK4	PLK1	FRAP1	FRAP1	PLK1	CCL14;CCL15	PIK3R4	RAF1	MAPKAPK5
PLK1	POLD1	PLK1	BUB1B	PLK4	MYO3A	FRAP1	MAP3K12	DGKH
AURKB	RPS6KB1	CDC2	CDK2	SMG1	PRKG2	CHEK1	TGFBR2	PTK2
PLK1;RPL37A	CABC1	MASTL	RAF1	MERTK	PLK1;RPL37A	RIOK2	RIPK2	LIMK1
PDPK1	BRD4	PRKAA1	KDR	CDK2	FRAP1	SMG1	DDR1	PLK4
CAMK4	LMTK3	DYRK1A	PLK1	PI4KB	EPHA5	PLK1;RPL37A	PRKCI	NTRK1
EPHA2	ADCK5	PLK1;RPL37A	MYO3B	MAPK15	BRD4	TGFBR1	CDKL2	CAMK2G
RPS6KA4	ADCK4	EGFR	ERBB3	BUB1B	SGK1	RAF1	CSNK1G2	ROR2
ALPK2	CDC2L1;CDC2L2	RPS6KA4	DHDDS	PRPF4B	HIPK2	ICK	WNK1	IRAK3
FYN	AATK	NPR1	RPS6KA4	PRKAA1	AURKB	AURKA		BRD2
TIE1	NEK8	VRK1	TAF1	FRAP1	CDK2	BUB1B		CDK9
ICK	STRADB	ROR2	BMPR1A	CRKRS	MST1R	MAP4K3		PRKACB
BUB1B	EEF2K	MELK	STK11	ERBB3	PIK3R4	MAP3K6		ATM;NPAT
POLD1	MAST3	TRIM24	TRRAP	CHEK1	WEE1	MYLK		
WEE1	RPS6KL1	SLTM	CDKL5	MAPKAPK5	SGK269	PCTK1		
CCL14;CCL15	TSSK2	MAP4K3	CDK5	SGK3	TIE1	EPHA2		
MAP3K7	MYO3B	CSNK1G2	MMAB	EPHA6	TEX14	ALPK2		
LATS2	PTK7	TIE1	PIK3C3	AURKB	RIPK3	PIK3C2G		
PRKX	NRBP2	MAP3K8	EPHA6	RIPK3	RAF1	STK32A		
BRD2	TYK2	IKBKB	SMG1	SLTM	RIOK2	KDR		
PRKAA2	ADRBK2	TYK2	PIK3CB	EGFR	MYLK	WNK1		
FRAP1	ICK	PRKD2	MAST3	CDKL3	ULK3	CLK2;CLK2P		
GSK3B	STK25	MAPK15	PI4KB	ATM;NPAT	PTK2	STK16		
CLK2;CLK2P	TRRAP	YES1	EIF2AK3	PDPK1	PRKDC	MAPK6		
CHEK1	ALPK2	FER	MARK3	HIPK1	SNF1LK2	DGKH		
TRRAP	INSRR	BUB1	CDC2	PCTK2	CLK2;CLK2P	NPR1		
EEF2K	CIT	KDR	MAPK8	PI4KA	TAF1	STK33		
PRKDC	RAC1	MMAB	CCL14;CCL15	PRKCI	PRKAA1	KIAA1804		
BMP2K	STK3	PDPK1	ANTXR1	TRIM33	NPR1	SNF1LK2		
EIF2AK3	CSNK1A1	TRRAP	PNCK	TRRAP	MAP3K12	EIF2AK3		
LIMK2	RIPK3	BTK	PAK2	MAP3K1		RP6		
TNK2	NPR1	PKN2	ROR2	ROR2		HIPK1		
HCK	VRK1	MAPK6	STK3	NTRK1		ATM		
ATR	TLK1	PRKX	CSNK1G2	MAP4K3		CDKL3		
TGFBR2	MAP3K10	PTK2	WNK1	RIOK2		CSNK1G2		
CDKL3	TAF1	MAP3K6	PDPK1	MELK		CCL14;CCL15		
CIT	MARK1	RIPK3	ABL2	DHDDS		MARK3		
NEK9	SCYL3	PRKCQ	PRKACA	CDKL4		PKN1		
LMTK3	STK32C	BRD2	EPHA10	ATR		ATM;NPAT		
PIM1	NEK7	ERBB3	CDKL4	PRKDC		TTK		
NR4A3	FYN	POLD1	WEE1	ALPK2		PASK		
VRK3	MAPK8	PKN1	MAPK10	RPS6KB1		CDC2		
BMPR1A	NTRK1	PI4KA	NEK8	PIK3CA		RORA		
NTRK1	KSR2	RAF1	PTK7	TRPM7		SNRK		
CSNK1D	NR4A3	PRKDC	LIMK2	SNF1LK2		RIPK3		
MAPK6	PCTK2	EPHA6	MAP2K5	WNK1		RIPK5		
PRKCQ	TRIM28	ANTXR1	PAK6	MAPK1		IRAK3		
TAF1	MKNK2	PRPF4B	STK32A	STK25				
ULK3	RPS6KA4	PRKACB	CSNK1A1	MLKL				
RAF1	PAK2	CLASP1	CLK2;CLK2P	PKN2				
PRPF4B	RIPK4	ATR	MYO3A	PRKCQ				
RIPK3	PKN2	CDK2	BRD2	WEE1				
MAP4K3	VRK3	KIAA1804	TTK	PRKACB				
MELK	KSR1	EPHB4	GPI	EPHA5				
JAK2	PIK3C2G	SMG1	ITK	MYLK				
IRAK2	MAP3K15	DMPK	HIPK1	EPHA2				
SNF1LK2	CDKL1	VRK2	MAP4K3	BRD4				
KSR1	MYO3A	STK35		PIK3R4				
PASK	MARK4	NEK7		STK39				
PTK7	HIPK4	PIK3CA		RPS6KA2				
ROCK1	PRKCG	MAPK1		MYO3B				
PTK6	ADCK1	CAMK2G		IKBKB				
TRIO	PINK1	STK31		RAF1				
CSNK1G2	HIPK1	ICK		PRKCZ				
CDK4	BRD2	BUB1B		ACVR1C				
DHDDS	DGKE	MAP2K3		MAPK8				
EPHB1	SCYL2	TAF1		RIOK1				
SLTM	GUCY2C	PCTK1		PKN1				
EPHB4	STK35	CAMK1G						
SCYL2	CDKL5							
MARK1	PI4KB							
CABC1	FASTK							
TRIM33	PKN1							
ERBB3	MASTL							
MKNK2	TRIB2							
MAPK8	PRKCQ							
LMTK2	BRAF;KIAA1549							
DYRK1B	CDC42BPG							
MYLK								
BUB1								
FGR								
ERBB2								
