# Supplementary figures and images for: ARID1A mutation sensitizes most ovarian clear cell carcinomas to BET inhibitors
Source: Oncogene. 2018 May 15;37(33):4611–25. doi: 10.1038/s41388-018-0300-6 (PMC6095834; doi:10.1038/s41388-018-0300-6)

Suppl. Figure 3

A

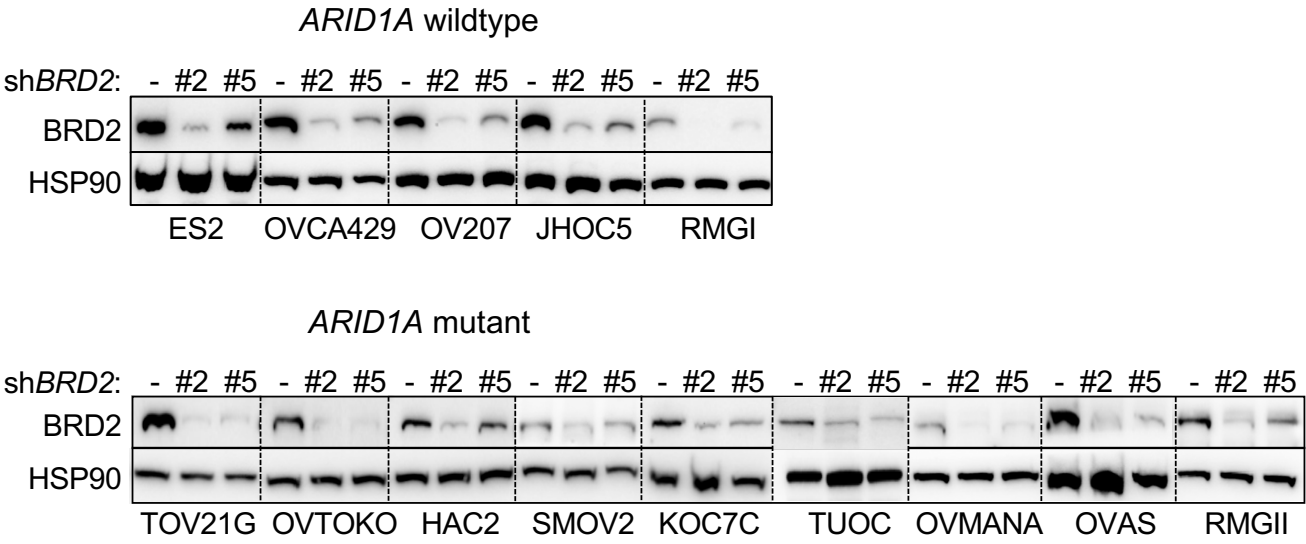

B

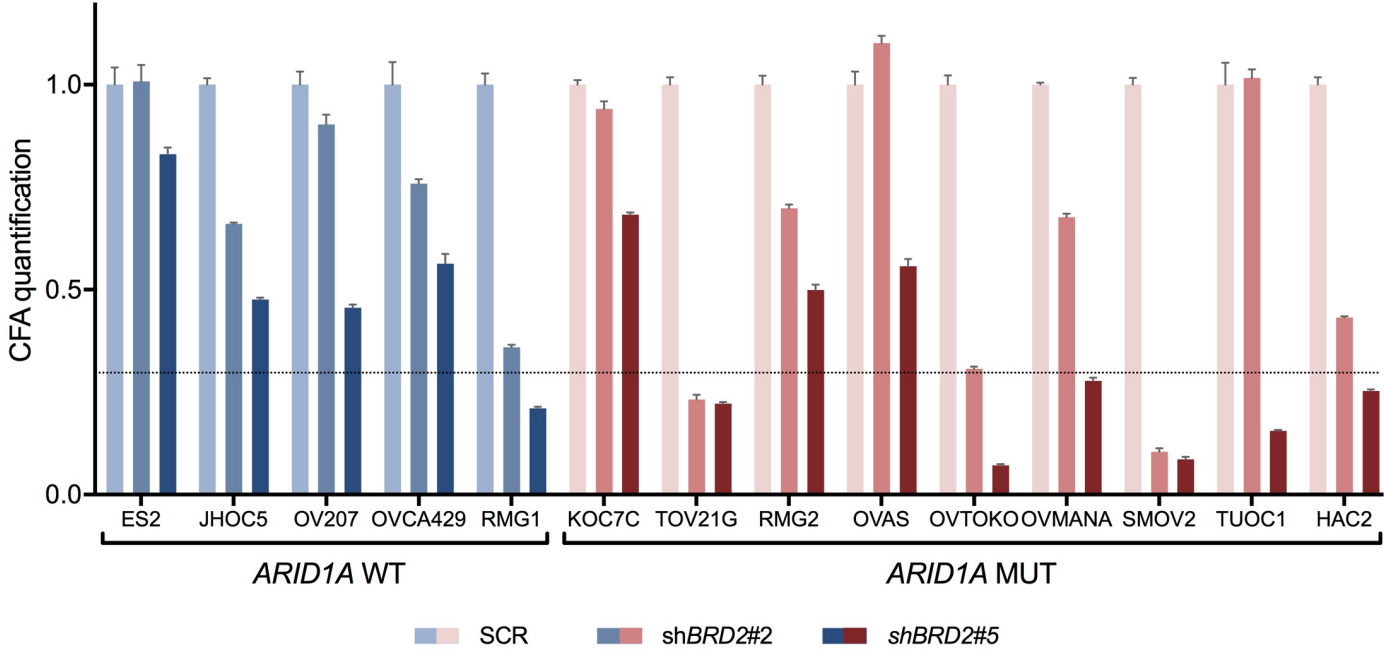

Supplement: Supplementary file 3 — Figure S3. BRD2 knockdown in the OCCC cell line panel [file 41388_2018_300_MOESM3_ESM.pdf]

Suppl. Figure 4

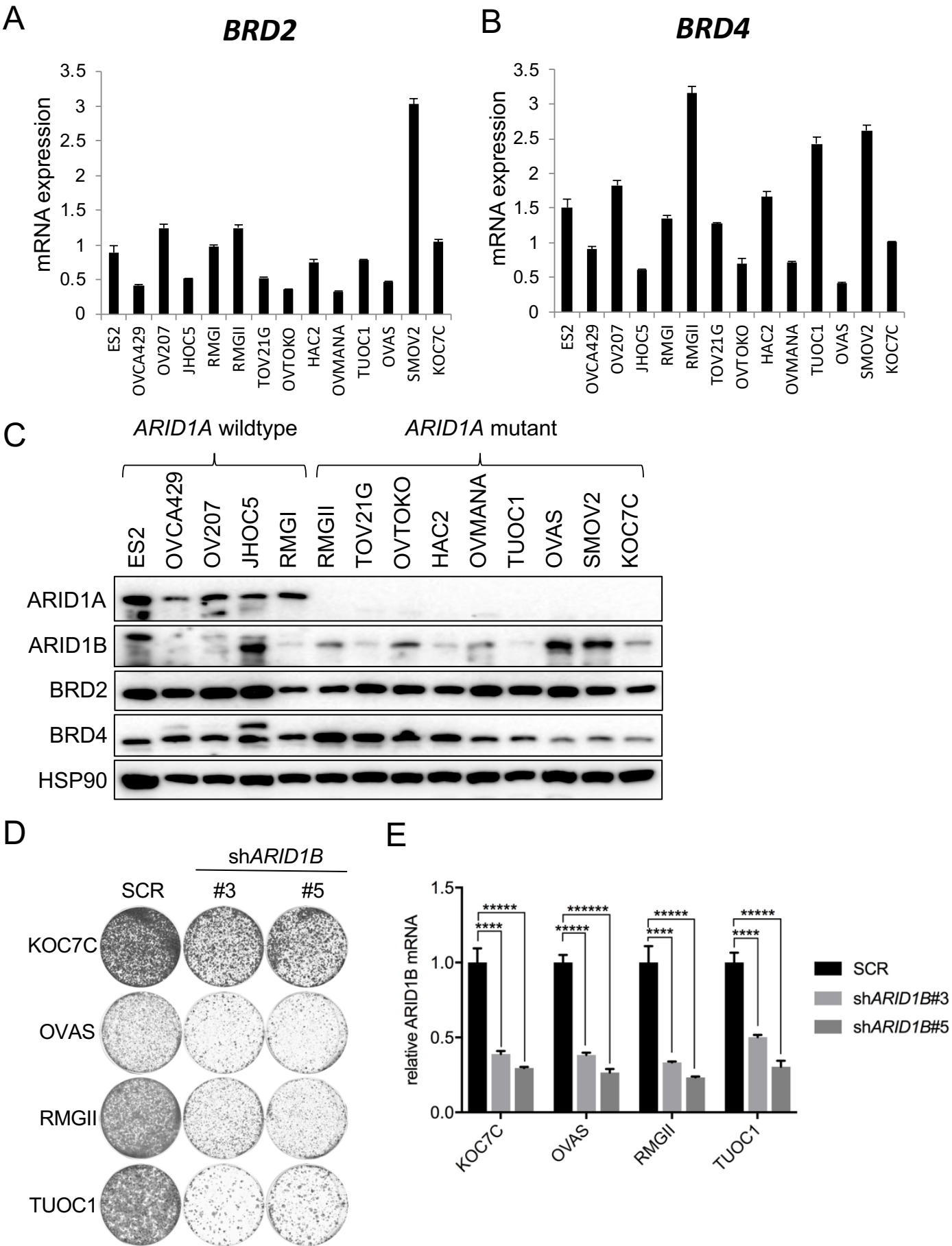

Supplement: Supplementary file 4 — Figure S4. BRD2 and BRD4 status OCCC cell line panel [file 41388_2018_300_MOESM4_ESM.pdf]

Suppl. Figure 5

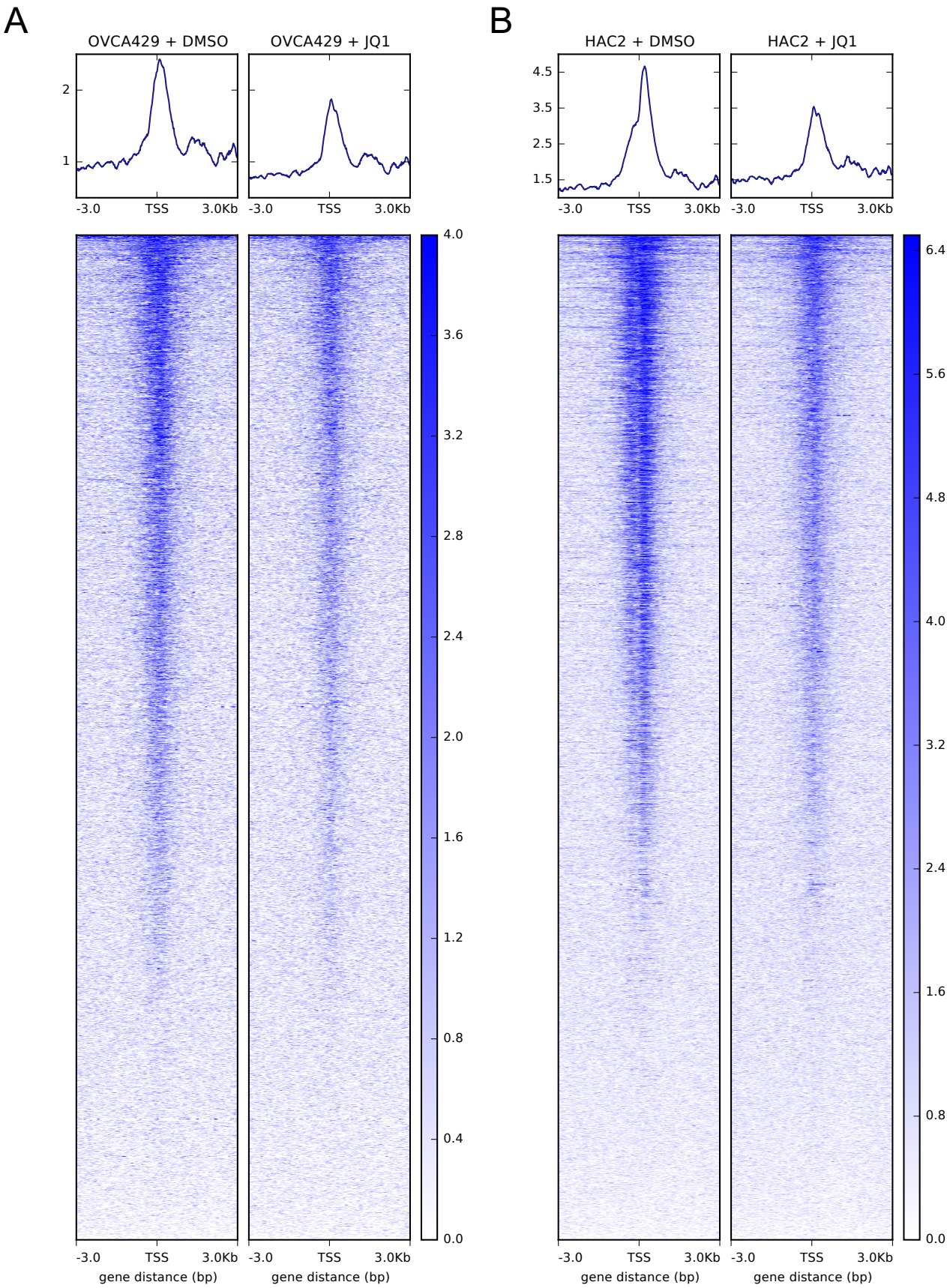

Supplement: Supplementary file 5 — Figure S5. BRD2 ChIP-seq heatmaps at TSS for OVCA429 and HAC2 [file 41388_2018_300_MOESM5_ESM.pdf]

Suppl. Figure 6

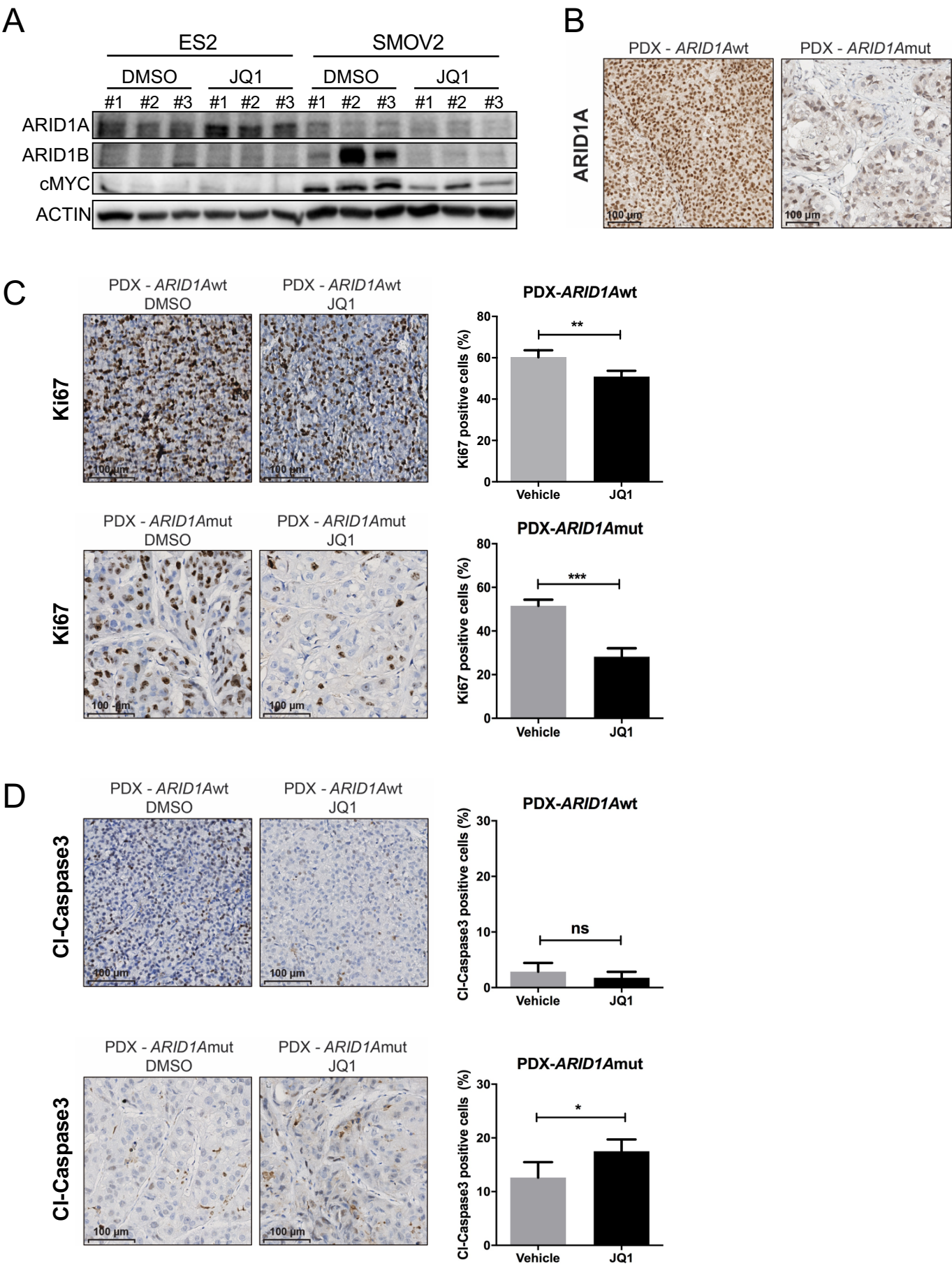

Supplement: Supplementary file 6 — Figure S6. ARID1B protein analysis and H&E/ Cleaved-caspase3 staining of JQ1 treated tumors [file 41388_2018_300_MOESM6_ESM.pdf]
